# A Single-Session, Web-Based Parenting Intervention to Prevent Adolescent Depression and Anxiety Disorders: Randomized Controlled Trial

**DOI:** 10.2196/jmir.9499

**Published:** 2018-04-26

**Authors:** Mairead C Cardamone-Breen, Anthony F Jorm, Katherine A Lawrence, Ronald M Rapee, Andrew J Mackinnon, Marie Bee Hui Yap

**Affiliations:** ^1^ Monash Institute of Cognitive and Clinical Neurosciences School of Psychological Sciences Monash University Melbourne Australia; ^2^ Melbourne School of Population and Global Health University of Melbourne Melbourne Australia; ^3^ Centre for Emotional Health Macquarie University Sydney Australia; ^4^ Black Dog Institute University of New South Wales Sydney Australia

**Keywords:** adolescent, mental health, depression, anxiety, parenting, family, preventive health services, Internet

## Abstract

**Background:**

Depression and anxiety disorders are significant contributors to burden of disease in young people, highlighting the need to focus preventive efforts early in life. Despite substantial evidence for the role of parents in the prevention of adolescent depression and anxiety disorders, there remains a need for translation of this evidence into preventive parenting interventions. To address this gap, we developed a single-session, Web-based, tailored psychoeducation intervention that aims to improve parenting practices known to influence the development of adolescent depression and anxiety disorders.

**Objective:**

The aim of this study was to evaluate the short-term effects of the intervention on parenting risk and protective factors and symptoms of depression and anxiety in adolescent participants.

**Methods:**

We conducted a single-blind, parallel group, superiority randomized controlled trial comparing the intervention with a 3-month waitlist control. The intervention is fully automated and consists of two components: (1) completion of an online self-assessment of current parenting practices against evidence-based parenting recommendations for the prevention of adolescent depression and anxiety disorders and (2) an individually tailored feedback report highlighting each parent’s strengths and areas for improvement based on responses to the self-assessment. A community sample of 349 parents, together with 327 adolescents (aged 12-15 years), were randomized to either the intervention or waitlist control condition. Parents and adolescents completed online self-reported assessments of parenting and adolescent symptoms of depression and anxiety at baseline, 1-month (parent-report of parenting only), and 3-month follow-up.

**Results:**

Compared with controls, intervention group parents showed significantly greater improvement in parenting risk and protective factors from baseline to 1-month and 3-month follow-up (F_2,331.22_=16.36, *P*<.001), with a small to medium effect size at 3-month follow-up (*d*=0.33). There were no significant effects of the intervention on adolescent-report of parenting or symptoms of depression or anxiety in the adolescents (all *P*>.05).

**Conclusions:**

Findings suggest that a single-session, individually tailored, Web-based parenting intervention can improve parenting factors that are known to influence the development of depression and anxiety in adolescents. However, our results do not support the effectiveness of the intervention in improving adolescent depression or anxiety symptoms in the short-term. Long-term studies are required to adequately assess the relationship between improving parenting factors and adolescent depression and anxiety outcomes. Nonetheless, this is a promising avenue for the translation of research into a low-cost, sustainable, universal prevention approach.

**Trial Registration:**

Australian New Zealand Clinical Trials Registry: ACTRN12615000247572; https://www.anzctr.org.au/Trial/Registration/TrialReview.aspx?ACTRN=12615000247572 (Archived by WebCite at http://www.webcitation.org/6v1ha19XG)

## Introduction

### Background

Depression and anxiety disorders are among the leading contributors to global burden of disease [1]. Importantly, these disorders have peak onset early in life, accounting for the greatest proportion of disability in young people (aged 12-24 years [2-4]). Early onset of these disorders is associated with deleterious sequelae across the lifespan, leading to substantial cost at individual, social, and economic levels [4,5]. Preventive efforts targeting these disorders early in life are therefore a global priority [4]. Given that the onset of these disorders peaks during adolescence, this is an ideal period to target such preventive efforts [4].

Due to their frequent comorbidity, overlap of symptoms, and shared aetiological factors, depression and anxiety disorders are often grouped under an *internalizing* cluster [6-8]. Transdiagnostic approaches to prevention may therefore enhance the efficacy and cost-effectiveness of preventive programs [6,8,9]. In particular, a growing body of evidence highlights the important role of families in the prevention of internalizing problems in children and adolescents. Many risk factors for depression and anxiety involve the family environment (eg, interparental conflict and family connectedness [9-11]) or parenting (eg, parental warmth, aversiveness, and over-involvement [9,12-14]). Other factors can be detected and responded to by parents (eg, negative affectivity, coping style, behavioral inhibition, and excessive reassurance seeking [8,15-17]), or are directly influenced or modeled by parents (eg, parental response to child emotions and modeling or reinforcement of anxious behaviours [18-21]). As these factors are potentially within parents’ control, they are amenable to preventive intervention. Furthermore, parents are a strategic target for preventive approaches for several reasons: most adolescents live with their parents, increasing proximity and exposure to preventive strategies; parents are intrinsically motivated to take actions to promote their child’s health [21]; and parents may have the foresight to appreciate the value of prevention [21]. Given these factors, it is unsurprising that the role of family and parenting in the promotion of youth mental health has been recognized as a key research translation priority [4,14,22,23].

Promisingly, existing research demonstrates that depression and anxiety disorders in children and adolescents can be prevented. A number of recent systematic reviews and meta-analyses support the efficacy of existing preventive programs (eg, [23,24-28]). Most of these programs are delivered directly to the child (eg, through schools), often utilizing a cognitive behavioral approach and delivered face-to-face by trained professionals (eg, [25,26-28]). In programs that do include a parent component, this often involves teaching parents how to understand and implement the content that is delivered to the child, rather than targeting specific parenting factors. Some exceptions to this include programs that target family conflict, parental over-involvement, or parent-child relationship as part of broader cognitive behavioral or psychoeducational prevention programs for parents of children at risk of anxiety or depression [29-34]. One program that aims to improve parent emotion socialization practices has also shown improvements in family conflict and internalizing symptoms in adolescents [35,36].

Additionally, a recent meta-analysis of preventive parenting interventions (ie, where the parent received >50% of the intervention) for internalizing symptoms in children and adolescents (aged 0-18 years) found lasting preventive effects from 6 months to 11 years postintervention [37]. Notably, of the 51 studies included in the review, only 3 targeted parents of adolescents. Thus, in comparison with programs for parents of younger children, there is a lack of preventive parenting interventions for internalizing disorders in adolescents. Although the amount of variance explained by individual parenting or family factors is small [9,38-40], targeting multiple factors in one intervention may result in larger effect sizes. Furthermore, preventive parenting programs could be used alongside existing preventive interventions (eg, school-based programs delivered to the child), thereby increasing the number of risk and protective factors targeted for each child or family.

Existing parenting interventions face many challenges in engaging parents because of barriers such as time and scheduling constraints, geographical distance, childcare provision, and financial cost [41-43]. In addition, mental health interventions are often associated with added concerns about privacy, the perception of being a “bad” parent, and stigma [44-46]. Online delivery is one potential strategy to overcome some of these barriers. Web-based programs reduce geographic and time constraint barriers, can be accessed privately and anonymously, and can be disseminated widely at low cost. Furthermore, research shows that parents are already seeking information about both parenting and mental health online [47-50]; therefore, this is an ideal space for delivery of preventive parenting programs. Moreover, there is evidence for the efficacy of online interventions for the treatment (eg, [51,52]) and prevention (eg, [53,54]) of depression and anxiety, as well as the delivery of parenting programs (eg, [31,55-57]).

### The Partners in Parenting Intervention

To address some of the gaps discussed above, our team recently developed the Partners in Parenting (PiP) intervention—a Web-based, multi-level public health approach that aims to support parents in prevention and early intervention for adolescent depression and anxiety disorders (see [58] for further details). The PiP intervention comprises three components: (1) a parenting self-assessment that assesses current parenting practices against evidence-based parenting recommendations for the prevention of adolescent depression and anxiety [59]; (2) an individually tailored feedback report based on each parent’s responses to the self-assessment, highlighting parenting strengths and areas for improvement; and (3) a set of interactive modules to support parents in applying the parenting recommendations. The development of PiP was guided by the persuasive systems design (PSD) model that aims to use technology to promote behavior change through principles such as tailoring, personalization, and suggestion [60]. The content of all components of PiP is based on the evidence-based parenting guidelines *How to Prevent Depression and Clinical Anxiety in Your Teenager: Strategies for Parents* (referred to henceforth as *the Guidelines* [61]). The Guidelines represent the synthesis of high-quality research evidence and international expert consensus. They were developed via a systematic review and meta-analysis of parenting risk and protective factors for adolescent depression and anxiety, published by researchers internationally [9], followed by a Delphi international expert consensus study of parenting strategies important for the prevention of depression and anxiety in adolescents [21]. The core content of the Guidelines (and subsequently PiP) should therefore be applicable to an international audience. However, as we initially developed PiP to trial with an Australian population, some aspects of the language and examples provided may need to be adapted for use in other English-speaking countries (eg, specific terms, types of health professionals, and links to additional local resources).

Preventive interventions can be universal (ie, delivered to all individuals regardless of level of risk), selective (ie, targeting individuals with known risk factors), or indicated (ie, targeting individuals showing signs or symptoms [62]). The PiP multi-level intervention is designed to support parents across the prevention continuum [58]. The first two components (parenting self-assessment and personalized feedback report) can serve as a stand-alone single-session intervention, which is most appropriate as a universal prevention approach [58]. This is likely to be an acceptable level of intervention for parents who are motivated, educated, and whose child does not have existing mental health problems [58]. The brevity of the single-session intervention may be particularly appealing to parents who are not willing or able to commit to the more intensive program (ie, series of interactive modules). As well as the Web programming ensuring intervention fidelity, the single-session intervention increases the likelihood of users achieving 100% adherence compared with interventions that require participants to return for multiple sessions. This is a notable advantage over many existing online interventions where adherence is often low [63,64]. Within PiP, parents can “step up” to the next level of intervention (interactive modules) based on need (ie, selective or indicated prevention) or parent preference (ie, parents who do not find a single-session intervention sufficient). The single-session intervention is also designed to serve as a prompt for parents to seek further assistance if required (eg, mental health support for themselves or their child or the PiP interactive modules).

Evaluation of the full PiP intervention is currently underway. Three-month postintervention results demonstrate that PiP can improve parenting risk and protective factors compared with an active control condition [65]. This paper evaluates the effectiveness of the single-session component of PiP as a stand-alone intervention. This is important given the proposed multi-level model (ie, some parents will only complete the single-session component), as well as the high attrition rates typically observed in online intervention trials (eg, [63,66,67]). As this is a preliminary trial of the newly developed intervention, and to align with a concurrent randomized controlled trial (RCT) of the full PiP intervention, this paper only examines the short-term (3-month) effects of the intervention. To our knowledge, the intervention evaluated in this paper is the first single-session, Web-based, tailored psychoeducation intervention targeting the wide range of parenting risk and protective factors for the development of depression and anxiety in adolescents.

### Study Aims and Hypotheses

The primary aim of this study was to evaluate the short-term effect of the intervention on evidence-based parenting risk and protective factors for adolescent depression and anxiety disorders, as assessed by parent-reported concordance with the Guidelines. The secondary aims of the study were to examine the effects of the intervention on parent and child reported symptoms of depression and anxiety in adolescent participants and adolescent report of parenting. To do this, we conducted an RCT comparing the intervention with a waitlist control condition. We hypothesized that compared with waitlist controls, intervention group parents would show a greater increase in concordance with the Guidelines from baseline to postintervention and follow-up. We also predicted a greater reduction in symptoms of adolescent depression and anxiety in the intervention compared with the control group.

## Methods

### Study Design and Participants

The trial was a single-blind, parallel-group, superiority RCT with two conditions: intervention and 3-month waitlist control. Assessments took place at baseline (pre-intervention), 1-month postintervention, and 3-month follow-up, with data collection from April 2015 to November 2016. Participants were parents or primary caregivers of at least one adolescent aged 12 to 15 years, who resided in Australia, had regular Internet access, and had an email account. Computer and Internet literacy was implicit in the eligibility criteria and registration process. Only one parent and one adolescent from each family was allowed to participate. No other exclusion criteria were specified. Parents were able to participate if their adolescent declined participation; therefore, not all parent participants had an associated adolescent participant. Recruitment was primarily via secondary schools across Australia, as well as online networks and social media. Schools were requested to distribute recruitment materials (flyers and participant explanatory statements) via their usual methods of communication with parents. This predominantly involved school newsletters (electronic and hard copy), online parent portals, and email communication. Hard copy flyers were also made available (eg, at parent information evenings). Other recruitment methods included an email to the mailing list of Mental Health First Aid Australia, a social media (Facebook and twitter) post by *beyondblue* (the Australian national depression and anxiety initiative), and an advertisement in an Australian parenting magazine (*Exploring Teens*, electronic and hard copy versions). A power analysis (conducted using Stata-based software [68]) indicated a required sample size of 294 parents and adolescents to detect a small effect size (Cohen *d*=0.20) with power of .80 and alpha of .05 for the primary outcome. To allow for approximately 15% attrition, we aimed to recruit 340 parents and adolescents. Our final sample comprised 349 parents together with 327 adolescents at randomization. The trial was approved by the Monash University Human Research Ethics Committee (approval number CF14/3886-2014002023) and prospectively registered with the Australian and New Zealand Clinical Trials Registry (registration number ACTRN12615000247572).

### Intervention

As discussed, the single-session intervention evaluated in this paper is the first component of the multi-level PiP program [58]. The intervention provides individually tailored psychoeducation to each parent. Parents first complete an online parenting scale that assesses their current parenting practices and beliefs against the recommendations in the Guidelines (the Parenting to Reduce Adolescent Depression and Anxiety Scale, PRADAS [59]). On the basis of their responses to the PRADAS, each parent receives an individually tailored feedback report highlighting parenting strengths and areas for improvement. The feedback report covers the nine domains of parenting in the Guidelines (see Table 1) and recommends specific actions parents could take to improve their concordance with the Guidelines (see Multimedia Appendix 1 for screenshots of the intervention). Feedback messages are designed to be brief, and links are provided for parents to seek further information if they wish. The intervention is fully automated and designed to tailor the content of the Guidelines for each parent, according to principles of the PSD model [56]. For example, feedback messages suggest specific actionable strategies that parents could implement (suggestion and tuneling principles), the content of the Guidelines is reduced to a shorter feedback report covering the areas deemed most relevant to each parent (reduction principle), feedback messages are tailored to each parent to increase relevance of the recommendations (tailoring principle), each feedback section includes praise for areas of strength (praise principle), and both the PRADAS and feedback report are personalized with the adolescent’s name and gender (personalization principle).

The development of the intervention included consultation with a reference group of parents of adolescents to ensure acceptability to target end users. This involved conducting three 2-hour workshops in which 22 parents of adolescents aged 11 to 18 years (19 mothers, 3 fathers; n=7-8 per workshop) were shown a draft of the PRADAS and feedback messages. Parents completed the PRADAS for themselves and read sample feedback reports before participating in facilitated discussion regarding ways to improve the intervention. Feedback from parents was incorporated into the final version of the intervention. This included changes to the language used, content of the feedback report, additional practical strategies or ideas on how to implement these, and the addition of a section at the beginning of the feedback report suggesting how parents may wish to work through the information provided.

### Measures

#### Primary Outcome Measure: The Parenting to Reduce Adolescent Depression and Anxiety Scale

The PRADAS is a self-report, criterion-referenced measure of parental concordance with the Guidelines [59]. The scale comprises a total of 73 items across eight subscales (parent-child relationship, involvement, family rules, home environment, health habits, dealing with problems, coping with anxiety, and professional help-seeking). One of the original nine subscales (relationships with others) was removed from the final version of the PRADAS because of its unsatisfactory psychometric properties. Most items assess specific recommendations in the Guidelines scored on a Likert-type frequency scale (never, rarely, sometimes, and often). Being a criterion-referenced measure, each item has a cut-off score for mastery, with items scored as either concordant (1) or nonconcordant (0) with the Guidelines. Items can be summed to form eight subscale scores and a total score, ranging from 0 to 73 (higher scores indicate greater concordance with the Guidelines). The total score has demonstrated high reliability, as measured by the agreement coefficient (.97), and acceptable one-month test-retest reliability (.78). It has also shown convergent validity with two other parenting measures and a small association with adolescent depression and anxiety symptoms [59]. We utilized the total score as it has the strongest psychometric properties. Reliability was high in our sample (agreement coefficient .97).

#### Secondary Outcome Measures

##### Parenting to Reduce Adolescent Depression and Anxiety Scale-Adolescent Report

The PRADAS-Adolescent report (PRADAS-A) was developed by our team to assess the adolescent’s perspective on the parenting factors assessed by the PRADAS. The original PRADAS items were reworded to reflect the adolescent’s perspective at a developmentally appropriate level. Items not applicable to the adolescent (eg, parental knowledge, attitudes, beliefs, or hypothetical questions) were not included. The PRADAS-A comprises 43 items across the same eight subscales as the PRADAS. As with the PRADAS, one original subscale (relationships with others) was removed from the final version of the scale. Response options and scoring are similar to the PRADAS; most items are on a Likert-type frequency scale (never, rarely, sometimes, and often) and are scored as either concordant (1) or nonconcordant (0) with the Guidelines. Higher scores indicate greater concordance with the Guidelines. Reliability has been shown to be high for the total score (agreement coefficient=.81) in a community sample of 670 adolescents aged 12 to 15 years [69].

**Table 1:** Parenting domains covered in the intervention, corresponding Guidelines topics, parenting risk or protective factors addressed, and example parenting recommendations.

Intervention domain	Guidelines topic	Risk or protective factors covered	Example recommended parenting strategy
Your relationship with [Child]^a^	Establish and maintain a good relationship with your teenager	Parental warmth, aversiveness, affection, emotional availability	Making time each day to ask [Child] about [his/her] day and what [he/she] has been doing, regardless of [his/her] response.
Your involvement in [Child]’s life	Be involved and support increasing autonomy	Parental over-involvement, autonomy granting, monitoring	Gradually increasing [Child]’s responsibilities and independence over time to allow [him/her] to mature.
[Child]’s relationships with others	Encourage supportive relationships	Parental encouragement of sociability	Take some time to talk through any social problems [Child] may have.
Your family rules	Establish family rules and consequences	Consistency of discipline	Noticing when [Child] behaves well, and rewarding [him/her] with positive consequences (eg, praise or privileges).
Your home environment	Minimize conflict in the home	Interparental conflict, parent-child conflict management, criticism, parental modeling of conflict management	Try not to argue with your partner if [Child] can hear. Frequent and intense conflict between parents increases a teenager’s risk of depression and clinical anxiety.
Health habits	Encourage good health habits	Diet, physical activity, sleep hygiene (7 items); responding to alcohol or drug use (5 items)	Set an example for [Child] by having good health habits (ie, healthy diet, regular exercise, and responsible use of alcohol) yourself.
Dealing with problems in [Child]’s life	Help your teenager to deal with problems	Problem solving, emotion regulation, stress management, modeling of problem solving approaches	When talking with [Child] about problems that [he/she] has dealt with, recognize and praise [his/her] problem-solving efforts (ie, what [he/she] did well when trying to solve the problem) rather than focusing on the outcome [he/she] achieved.
Coping with anxiety	Help your teenager to deal with anxiety	Anxiety management (avoidance, exposure), modeling of anxiety, management strategies	Try not to step in to help [Child] at the first sign of any stress or anxiety, as the way you respond to [Child]’s anxiety may unintentionally increase [his/her] anxiety. Instead, let [him/her] try to manage the situation [himself/herself] and provide help if [he/she] asks you to or if the anxiety persists.
Getting help with needed	Encourage professional help-seeking when needed	Professional help-seeking knowledge and behaviors (parent and child)	If you do notice a persistent change in [Child]’s mood or behavior: try to determine whether the change in mood or behavior is caused by a temporary situation or a more ongoing problem.

^a^Square brackets denote personalization with the adolescent’s name and gender.

The scale also has good 3-month test-retest reliability (.81) and moderate correlations with child-report symptom measures of depression and anxiety (*r*=.45 and .31, respectively [69]). The correlation between the PRADAS and PRADAS-A in the community sample was significant but small (*r*=.25; [69]). In the current sample, reliability of the total score was high (agreement coefficient=.81), and test-retest reliability (waitlist control group, baseline to 3 months) was good (.81). The correlation between PRADAS and PRADAS-A at baseline was .25 (*P*<.001).

##### Spence Children’s Anxiety Scale

The Spence Children’s Anxiety Scale (SCAS) is a widely used child- and parent-report measure of child anxiety [70,71]. The SCAS-Child report (SCAS-C) comprises 45 items, including six nonscored filler items, whereas the SCAS-Parent report (SCAS-P) has a total of 39 items. Items examine the degree to which the child experiences specific anxiety symptoms on a 4-point frequency scale (never, sometimes, often, and always). Items are scored from 0 (never) to 3 (always) and can be summed to form six subscale scores and a total anxiety score (ranging from 0-114, with higher scores indicating greater anxiety symptomology). We utilized the total anxiety score, which had high reliability in our sample, as measured by coefficient omega (a less biased reliability index than Cronbach alpha [72,73]; SCAS-C: omega=95; SCAS-P: omega=.93). The correlation between baseline SCAS-C and SCAS-P total scores in the current sample was .44 (*P*<.001).

##### Short Mood and Feelings Questionnaire

The Short Mood and Feelings Questionnaire (SMFQ) is a Child-report (SMFQ-C) and Parent-report (SMFQ-P) measure of depressive symptoms in children and adolescents [74]. The 13 items assess frequency of depressive symptoms in the previous 2 weeks on a 3-point scale of not true (0), sometimes true (1), or true (2). Items are summed to form a total score (ranging from 0-26, with higher scores indicating higher symptom levels). Reliability was high in our sample (omega=.92 for SMFQ-C and .91 for SMFQ-P). The correlation between baseline SMFQ-C and SMFQ-P scores was .47 (*P*<.001).

#### Process Evaluation: Parent Use and Satisfaction With the Intervention

Parents in both groups were asked five questions immediately postintervention (ie, after receiving their feedback at baseline or 3-month follow-up for the intervention and control groups, respectively). The questions were scored on Likert-type scales (see Multimedia Appendix 2) and assessed: (1) how much of the feedback was read, (2) satisfaction with the feedback, (3) perceived usefulness of the feedback, (4) intention to change based on the feedback provided, and (5) confidence in ability to implement the recommended changes. Additionally, at 1-month and 3-month follow-up, parents were asked whether they had attempted to make changes to their parenting since the previous assessment and their perceived success in making these changes. Parents were also asked if they had accessed any additional parenting resources or sought professional help for their own or their child’s mental health since the last assessment.

### Procedure

#### Registration and Consent

Parents self-selected by responding to advertisements and registering themselves and their adolescent via the publicly accessible trial website. Parents created an account with their email address and self-selected password and were required to verify their account via an account activation link sent to their email. Parents provided online consent and contact details for the adolescent; however, they were informed that they could still participate if their adolescent declined. Adolescents were then contacted by phone to explain the study requirements and obtain verbal assent. Adolescents were informed that their decision to participate or not would not affect their parent’s participation. Multimedia Appendices 3-5 present the participant informed consent information.

#### Baseline Assessments

All assessments were completed online via a dedicated trial website, and participants were required to log in with their username and password. Adolescents were guided through completion of their baseline assessment over the phone, with assistance provided by a member of the research team as necessary. The adolescent baseline assessment included the PRADAS-A, the SCAS-C, and the SMFQ-C. Adolescents were reimbursed with a $10 AUD e-voucher for completion of the assessment. Submission of the adolescent baseline assessment triggered an automated email sent to parent participants containing the link to their baseline assessment, which included the PRADAS, the SCAS-P, and the SMFQ-P. In cases where the adolescent did not participate, the research team cancelled the adolescent assessment, which triggered the automated email to be sent to the parent participant.

#### Randomization and Blinding

Upon completion of the parent baseline assessment, parents were randomly allocated to the intervention or control group via a computer-generated unblocked, unstratified randomization procedure on a 1:1 ratio. Parents were not blinded to their allocation. As assessments were conducted entirely online, assessor was not relevant. Researchers who phoned adolescent participants were blinded to allocation.

#### Intervention

Immediately following completion of the baseline assessment, intervention group parents were shown their individually tailored feedback on screen. They were also emailed the feedback report and a copy of the Guidelines in PDF format. Parents in the waitlist control group were informed via a website message that they would receive the intervention in 3 months’ time.

#### Follow-Up Assessments

Follow-up assessments were conducted with parents at 1-month postintervention and with both parents and adolescents at 3-months postintervention. Parents were sent an email containing the link to their online assessment, and adolescents were contacted via phone to guide them through completion of the assessment. For all assessments, parents who had not completed their assessment received reminder emails 7 and 14 days following the initial invitation and a phone call or text message 21 days after initial invitation.

##### One-Month (Postintervention) Follow-Up

Thirty days after baseline, parents were sent the invitation to complete their postintervention assessment that included the PRADAS to assess for changes in parenting. One month was chosen to allow sufficient time to read the feedback and attempt to implement the recommended changes. Mean duration to follow-up was 41.47 days (SD 10.76).

##### Three-Month Follow-Up

At 3-month follow-up, both parents and adolescents completed their respective versions of the PRADAS, SCAS, and SMFQ. Parents in the waitlist control group received their individually tailored feedback report and a copy of the Guidelines immediately following submission of their 3-month follow-up assessment. Parents and adolescents were reimbursed with a $10 AUD e-voucher each on completion of the assessment. Mean duration to follow-up was 102.74 days (SD 16.51) for parents and 96.14 days (SD 13.81) for adolescents.

#### Symptom Elevation Procedure

At baseline and 3-month follow-up (ie, when the SCAS and SMFQ were administered), participants who reported elevated adolescent symptoms on the SCAS and/or SMFQ were contacted. For the SCAS, elevated symptom status was defined as ≥1.5 SDs above the mean based on published Australian community sample norms [75]. As there are no published norms or consistent cut-off scores for the SMFQ, we considered a score of ≥8 on the SMFQ-C or SMFQ-P as elevated (based on the original SMFQ-C cut-off suggested by the developing authors [74]). In cases where both the child and parent reported elevation on either or both scales, parents were notified via email and encouraged to seek professional assistance for the adolescent as appropriate (n=38 at baseline, no significant differences between groups). All participants were provided with a list of websites and potential referral sources at baseline, and participants who reported elevated symptoms were encouraged to consult this. In cases where the child reported extreme elevation on the SMFQ (score > 20), a graduate clinical psychology student also phoned the adolescent to conduct a risk assessment and provide referral information as required (n=29 at baseline, no significant differences between groups).

### Statistical Analysis

Missing data rates were low (<1%) at both the item and participant level for all measures. Item-level missing data were replaced with subscale mean imputation for cases with less than 20% missing on a given measure. This is considered an acceptable approach for this amount of missing data [76]. Cases with greater than 20% missing on a measure were considered missing entirely from the analyses.

All analyses were conducted with an a priori type I error rate of .05. Independent samples *t* tests (for continuous variables) and chi-square tests (for categorical variables) were conducted to examine differences between completers and non-completers on outcome measures and participant characteristics. We also assessed for differences between complete parent-adolescent dyads and parents who participated alone.

Primary and secondary outcomes were analyzed with Mixed effect Model Repeated Measures (MMRM) analyses using the MIXED procedure of SPSS version 23 (IBM Corp) with an unstructured covariance matrix. MMRM is consistent with intention-to-treat principles, using all available data from each participant, including those who did not complete follow-up assessments [77]. This is a preferred analytic method for repeated measures designs as it yields unbiased results when data are missing at random and accounts for correlations between repeated measurements of the same participant [78]. For the SCAS and SMFQ, the distribution of model residuals violated the assumption of normality. We therefore applied log transformations to these variables, which improved the distribution of residuals. We ran the MMRM analyses on both the raw and transformed data. As the results did not change substantially and conclusions remained the same, we have reported results based on the raw data.

Finally, as the majority of parents were female, and there were more fathers in the control compared with intervention group (21 vs 7), we conducted sensitivity analyses by running all primary and secondary outcome analyses with data from mothers only. Similarly, as there were differences in participant characteristics between complete parent-adolescent dyads and parents who participated alone, we also ran all analyses using data from complete dyads only.

## Results

### Participant Characteristics

A total of 349 parents and 327 adolescents registered and completed baseline assessments. Parents had a mean age of 45.11 years (SD 6.11), and the majority were female (320/349, 91.7%). Adolescents had a mean age of 13.60 years (SD 1.03), with 49.2% (161/327) female. Table 2 presents baseline participant characteristics and Table 3 presents participant mental health characteristics. Mean scores on the SCAS-C and SCAS-P were within one SD of published Australian community sample norms for the age range of our sample [75]. Mean SMFQ scores were within one SD of other nonclinical samples (eg, [74,79,80]) and below clinical cut-off scores suggested by the developing authors (SMFQ-C score of 8 [74]) and other authors (eg, SMFQ-C score of 11 [79] and SMFQ-P score of 9 [81]). Mean SFMQ-C scores in our sample were also similar to those reported in large community sample of Australian and American adolescents [79]. Symptom levels in our sample are therefore considered to be within the normal range for this age.

### Comparison of Complete Parent-Adolescent Dyads and Parent-Only Participants

We compared participant characteristics and baseline parent-report measures between complete dyads (ie, both parent and adolescent participated at baseline; 93.7%, 327/349) and parents who participated without their adolescent. Parent-only participants had slightly older children (mean=14.1 vs 13.6 years, *t*_347_=2.38, *P*=.02), higher baseline SCAS-P and SMFQ-P scores (SCAS-P: mean=25.59 vs 16.17, *t*_21.71_=2.08, *P*=.049; SMFQ-P: mean=7.59 vs 4.06, *t*_22.01_=2.27, *P*=.03), were more likely to speak a language other than English at home (22.7% vs 7.6%; χ^2^_1_[N=349]=5.97, *P*=.02), and were more likely to report that their child had a history of or current anxiety disorder or concern regarding a previous undiagnosed mental health problem (all *P*<.05). This suggests that adolescents may have declined to participate because of their mental health or English proficiency. There were no differences for any other demographic variables, and complete dyads were balanced between groups (intervention group: 93.9%, 154/164; control group: 93.5%, 173/185; χ^2^_1_[N=349]=0.22, *P*=.88). As we intentionally allowed parents to participate without their adolescent to attain a more diverse sample, we kept data from parent-only participants in our main analyses. See sensitivity analyses below for comparison of results with complete dyads only.

### Attrition

**Figure 1:**
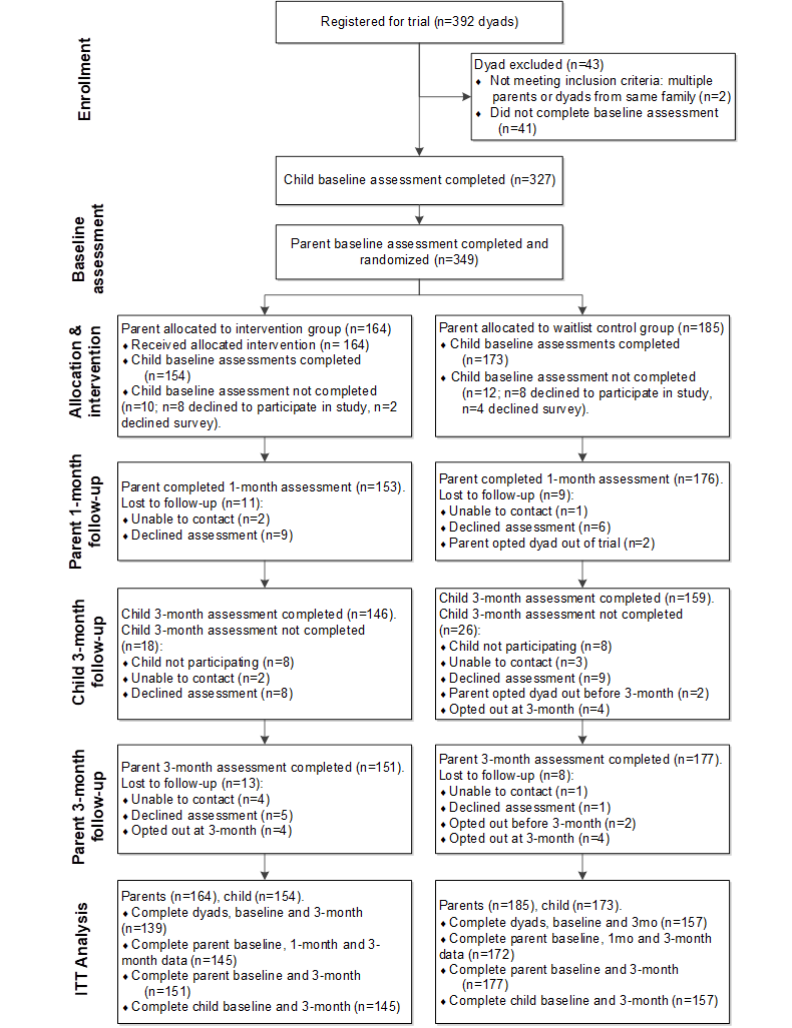
Consolidated Standards of Reporting Trials (CONSORT) participant flow diagram. ITT: intention-to-treat.

Figure 1 presents the participant flow diagram. Overall, the attrition rate was low at 6.0% (n=21) for parents (intervention group: n=13, 7.9%; control group: n=8, 4.3%) and 6.7% (n=22) for adolescents (intervention group: n=8, 5.2%; control group: n=14, 8.1%) at 3-month follow-up. Chi-square tests indicated no significant differences in attrition between groups at any time point (all *P*>.05). Chi-square tests and *t* tests were conducted to assess for differences in demographic characteristics and outcome measures between participants who completed and those who withdrew. There were no significant differences for any demographic variables (all *P*>.05). There were also no differences in scores on the PRADAS-A, SCAS-C, SCAS-P, or SMFQ-C. Significant differences were found for baseline PRADAS and SMFQ-P scores. Specifically, intervention group parents who withdrew from the 1-month follow-up assessment scored significantly lower on the baseline PRADAS than those who completed 1-month follow-up. This was not the case at 3-month follow-up or for control group parents. Similarly, intervention group parents who did not complete the 3-month assessment reported higher baseline SMFQ-P scores than those who did complete 3-month follow-up.

**Table 2:** Participant characteristics at baseline.

Parent or child characteristic		n (%)
**Parent relationship to child**		
	Mother	315 (90.3)
	Father	28 (8.0)
	Other^a^	6 (1.7)
**Parent relationship status**		
	Married or defacto	271 (77.7)
	Separated or divorced	58 (16.6)
	Single	16 (4.6)
	Widowed	4 (1.1)
**Family situation**		
	Intact family, child living with both parents	240 (68.8)
	Separated parents, shared care	36 (10.3)
	Child living with one parent (participant)	61 (17.5)
	Child living with one parent (not participant)	6 (1.7)
	Other	6 (1.7)
**Parent employment status**		
	Working full time	159 (45.6)
	Working part time	155 (44.4)
	Unemployed	35 (10.0)
**Parent study status**		
	Studying full time	57 (16.3)
	Studying part time	11 (3.2)
	Not studying	281 (80.5)
**Parent education level**		
	Secondary school year 7-12	27 (7.7)
	Trade or apprenticeship	1 (0.3)
	Technical and further education certificate or other technical qualification	40 (11.5)
	Diploma	61 (17.5)
	Bachelor degree	105 (30.1)
	Postgraduate	115 (33.0)
Language other than English spoken at home		30 (8.6)
**Parent Indigenous status**		
	Yes	4 (1.1)
	No	340 (97.4)
	Prefer not to say	5 (1.4)
**State of residence**		
	New South Wales	113 (32.4)
	Victoria	74 (21.2)
	Queensland	61 (17.5)
	Tasmania	49 (14.0)
	Australian Capital Territory	22 (6.3)
	South Australia	16 (4.6)
	Western Australia	13 (3.7)
	Northern Territory	1 (0.3)

^a^Other parent-child relationship category includes step-mother, step-father, grandmother, and legal guardian.

### Intervention Use

As the intervention was a once-off personalized feedback report, all parents allocated to the intervention group received the intervention. Following presentation of their feedback report, parents were asked how much of the feedback they had read. Of the 128 (78.0%) intervention group parents who answered this question, 102 (79.7%) reported reading all of it, 24 (18.8%) reporting reading about half of it, and 2 (1.6%) stated that they would read it later.

### Primary Outcome Measure: Parenting to Reduce Adolescent Depression and Anxiety Scale

**Figure 2:**
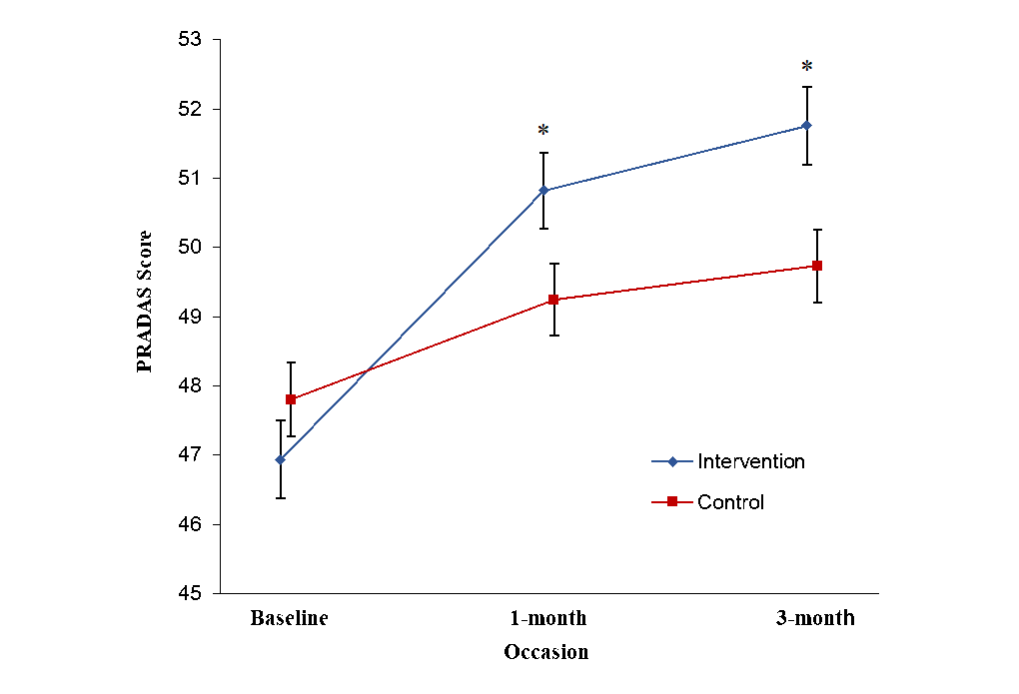
Estimated marginal means for Parenting to Reduce Adolescent Depression and Anxiety Scale (PRADAS) scores at baseline (n=349), 1-month (n=329), and 3-month (n=328) follow-up, estimated under group-by-measurement-occasion mixed model. Error bars represent standard errors. *Planned contrast significant at P<.001 level.

**Figure 3:**
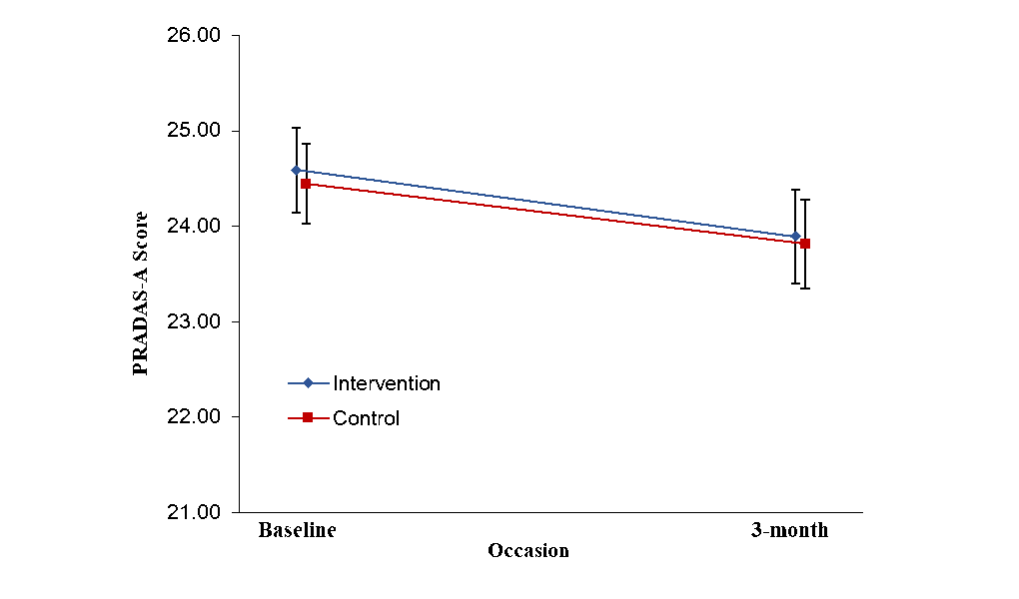
Estimated marginal means of Parenting to Reduce Adolescent Depression and Anxiety Scale-Adolescent report (PRADAS-A) scores from baseline to 3-month follow-up, estimated under group-by-measurement-occasion mixed model. Error bars represent standard errors.

Observed scores for the PRADAS at each occasion are presented in Multimedia Appendix 6 (Table 1). Figure 2 presents the estimated marginal means of PRADAS scores at baseline, 1-month, and 3-month follow-up, estimated under the group-by-measurement-occasion mixed model. The overall group-by-measurement-occasion interaction effect was significant (*F*_2,331.22_=16.36, *P*<.001), indicating a different pattern of change over time between groups. Planned contrasts between groups at each occasion indicated a significantly greater increase in PRADAS scores in the intervention compared with control group at both 1-month and 3-month follow-up (1-month follow-up, *t*_1,332.13_= 5.27, *P*<.001; 3-month follow-up, *t*_1,339.10_=4.87, *P*<.001). Effect sizes were small to medium (1-month follow-up, *d*=0.30 [95% CI 0.06-0.50]; 3-month follow-up, *d*=0.33[95% CI 0.05-0.49]). Figure 3 presents estimated marginal means of PRADAS-A scores from baseline to 3-month follow-up, estimated under the group-by-measurement-occasion mixed model.

At 1-month and 3-month follow-up, parents in both groups were also asked whether they had tried to make any changes to their parenting since the previous assessment time point and their perceived success in making changes. Chi-square analyses of the postintervention (1-month) assessment revealed a significant difference in attempts to change parenting (χ^2^_3_[N=307]=19.65, *P*<.001), with significantly more intervention group parents reporting making some changes to their parenting. This finding aligns with the parent-report of specific parenting behaviors on the PRADAS. There was, however, no significant difference between groups for parent-reported success in making changes to their parenting, χ^2^_3_(N=307)=6.26, *P*=.10. At 3-month follow-up, there were no significant group differences in reported attempts to change parenting (χ^2^_3_[N=323]=6.03, *P*=.11) or perceived success in making changes (χ^2^_3_[N=313]=6.03, *P*=.11). The proportion of parents who reported accessing additional parenting resources, or seeking professional help for their own or their child’s mental health, did not differ between groups at either 1-month or 3-month follow-up (all *P*>.05).

### Secondary Outcome Measures

Observed values for all secondary outcome measures are presented in Multimedia Appendix 6 (Table 2). Table 4 presents the estimated marginal means and MMRM group-by-measurement-occasion interaction results for these measures.

#### Parenting to Reduce Adolescent Depression and Anxiety Scale-Adolescent Report

The group-by-measurement-occasion interaction effect for PRADAS-A was nonsignificant (*F*_1,303.82_=0.02, *P*=.88), demonstrating no difference in the pattern of change over time between groups. A significant main effect for time was observed (*F*_1,303.82_=8.49, *P*=.004), with planned contrasts showing a significant reduction in PRADAS-A scores from baseline to 3-month follow-up for both groups (intervention, *P*=.04; control, *P*=.046).

#### Adolescent Symptoms

**Figure 4:**
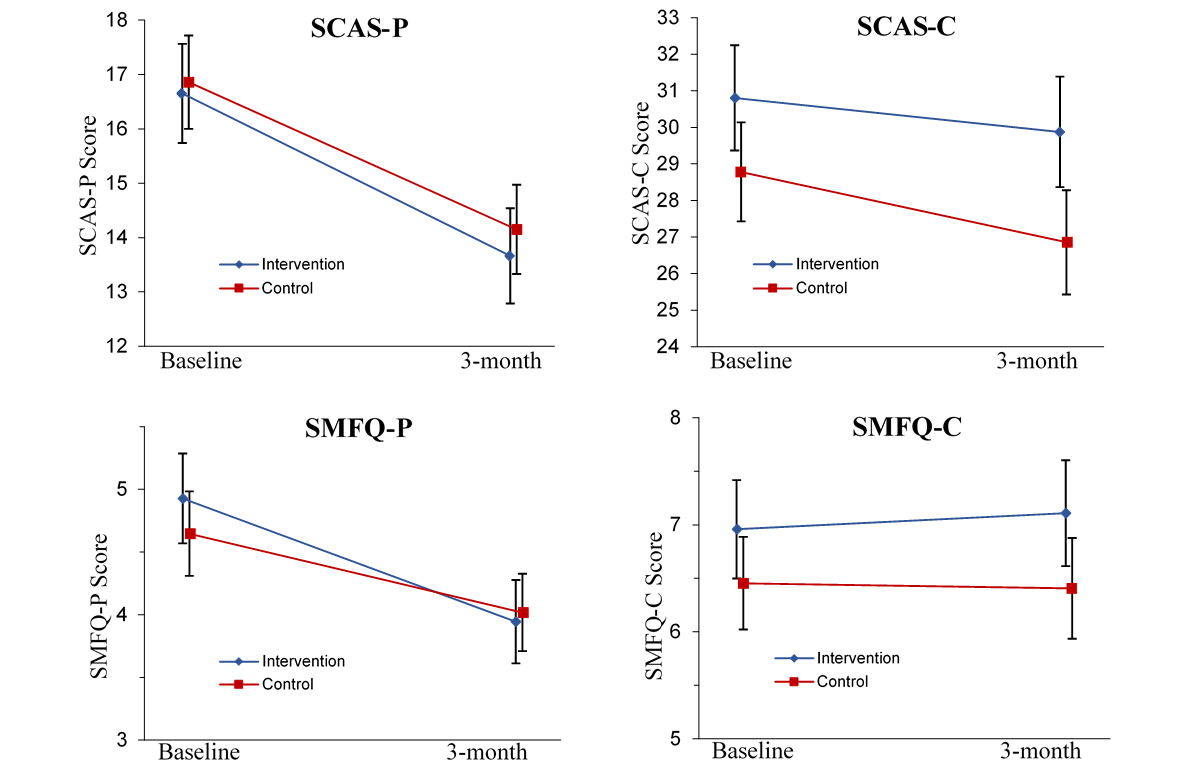
Estimated marginal means for Spence Children’s Anxiety Scale-Parent report (SCAS-P), Spence Children’s Anxiety Scale-Child report (SCAS-C), Short Mood and Feelings Questionnaire-Parent report (SMFQ-P), and Short Mood and Feelings Questionnaire-Child report (SMFQ-C) from baseline to 3-month follow-up, estimated under group-by-measurement-occasion mixed model. Error bars represent standard errors.

As shown in Table 4, the group-by-measurement-occasion interaction effects were not significant for any of the symptom measure analyses (all *P*>.05), suggesting no significant difference between the groups over time. Significant main effects for time were found for the SMFQ-P (*F*_1,324.76_=12.19, *P*<.001), SCAS-P (*F*_1,325.55_=70.54, *P*<.001), and SCAS-C (*F*_1,302.74_=5.55, *P*=.02), with planned contrasts demonstrating a reduction in symptoms for both groups from baseline to 3 months. There were no significant main effects for the SMFQ-C (all *P*>.05). Figure 4 presents the estimated marginal means for all symptom measures from baseline to 3-month follow-up.

**Table 3:** Parent and adolescent mental health characteristics, Spence Children’s Anxiety Scale (SCAS), and Short Mood and Feelings Questionnaire (SMFQ) scores at baseline. P and C indicate parent and child report, respectively.

Parent or child characteristic		Statistics
**Parental concern about child’s risk of developing depression, n (%)**		
	Not at all	77 (22.1)
	A little	175 (50.1)
	Yes	68 (19.5)
	Very much so	27 (7.7)
	Missing (declined to answer)	2 (0.6)
**Parental concern about child’s risk of developing an anxiety disorder, n (%)**		
	Not at all	79 (22.6)
	A little	165 (47.3)
	Yes	74 (21.2)
	Very much so	29 (8.3)
	Missing (declined to answer)	2 (0.6)
**Parental history or current mental health problem (as reported by parent), n (%)**		
	None	145 (41.5)
	Yes, past history	138 (39.5)
	Yes, current	33 (9.5)
	Yes, past and current	31 (8.9)
	Missing (declined to answer)	2 (0.6)
**Child history of mental health problem or behavioral disorder diagnosis (as reported by parent), n (%)**		
	None	231 (66.2)
	Depression	2 (0.6)
	Any anxiety disorder	10 (2.9)
	Autism spectrum disorder (including Asperger syndrome)	6 (1.7)
	Other mental health or behavioral disorder^a^	9 (2.6)
	Multiple diagnoses	11 (3.2)
	No formal diagnosis; however, I believe my child has experienced some emotional or behavioral problems	76 (21.8)
	Missing (declined to answer)	4 (1.1)
**Child current mental health or behavioral problems (as reported by parent), n (%)**		
	None	243 (69.6)
	Depression	1 (0.3)
	Any anxiety disorder	18 (5.2)
	Autism spectrum disorder (including Asperger syndrome)	7 (2.0)
	Other mental health or behavioral disorder^a^	7 (2.0)
	Multiple diagnoses	16 (4.6)
	No formal diagnosis; however, I believe my child is currently experiencing some emotional or behavioral problems	51 (14.6)
	Missing (declined to answer)	6 (1.7)
**Baseline symptom measures, mean (SD)**		
	SCAS-P score (n=349)	16.76 (11.66)
	SCAS-C score (n=326)	29.78 (17.87)
	SMFQ-P score (n=349)	4.28 (4.58)
	SMFQ-C score (n=325)	6.18 (5.66)

^a^This category includes attention-deficit/hyperactivity disorder, oppositional defiant disorder, conduct disorder, learning difficulties, or any other disorder specified by parents.

**Table 4:** Estimated marginal means, standard errors, and Mixed effect Model Repeated Measures (MMRM) test of group-by-measurement-occasion interaction for all secondary outcome measures. PRADAS-A: Parenting to Reduce Adolescent Depression and Anxiety Scale-Adolescent report; SCAS-C: Spence Children’s Anxiety Scale-Child-report; SCAS-P: Spence Children’s Anxiety Scale-Parent-report; SMFQ-C: Short Mood and Feelings Questionnaire-Child-report; SMFQ-P: Short Mood and Feelings Questionnaire-Parent-report.

Measure and occasion		Estimated marginal means (SE), n		MMRM group-by-measurement-occasion interaction effect			Cohen *d*^a^ (95% CI)
		Intervention	Control	*F*	Degrees of freedom	*P* value	
**PRADAS-A**				0.02	1,303.82	.88	
	Baseline	24.59 (0.44), 154	24.45 (0.42), 173				
	3-month	23.89 (0.49), 146	23.82 (0.47), 159				0.00 (−0.22 to 0.23)
**SCAS-P**				0.17	1,325.55	.68	
	Baseline	16.65 (0.91), 164	16.86 (0.86), 185				
	3-month	13.66 (0.88), 151	14.15 (0.82), 177				−0.06 (−0.29 to 0.15)
**SCAS-C**				0.67	1,302.74	.41	
	Baseline	30.81 (1.44), 153	28.78 (1.35), 173				
	3-month	29.88 (1.51), 145	26.86 (1.43), 159				0.18 (−0.05 to 0.40)
**SMFQ-P**				0.59	1,324.76	.44	
	Baseline	4.43 (0.36), 164	4.15 (0.34), 185				
	3-month	3.44 (0.33), 151	3.52 (0.31), 177				−0.04 (−0.26 to 0.17)
**SMFQ-C**				0.18	1,301.25	.68	
	Baseline	6.46 (0.46), 152	5.96 (0.43), 173				
	3-month	6.61 (0.50), 146	5.91 (0.47), 159				0.12 (−0.10 to 0.35)

^a^Cohen *d* effect size calculated based on observed means at end point (3-month follow-up) and SD of the control group.

#### Parent Satisfaction and Acceptability of the Intervention

Frequencies for parental responses to the process questions asked immediately postintervention (ie, after receiving their feedback at baseline or 3-month follow-up, for the intervention and waitlist control groups, respectively) are presented in Multimedia Appendix 2. Of the parents who answered these questions, the majority (93.6%) reported that they were somewhat or very satisfied with the feedback received, and 95.1% reported that they found the feedback either somewhat, very, or extremely useful. Most parents (90.2%) also reported that they were somewhat or very likely to change their parenting based on the feedback provided, although fewer parents were confident to do so (84.1% either very or moderately confident).

### Sensitivity Analyses

All results from primary and secondary outcome analyses using data from mothers only, as well as data from complete parent-adolescent dyads only, were consistent with results reported above.

## Discussion

### Principal Findings

This study aimed to evaluate the short-term effects of a single-session, Web-based, tailored psychoeducational parenting intervention on parenting factors and symptoms of depression and anxiety in adolescents. Results provide preliminary support for the effectiveness of the intervention for improving evidence-based parenting risk and protective factors for adolescent depression and anxiety disorders. In support of our first hypothesis, parents in the intervention group demonstrated a greater increase in self-reported concordance with the Guidelines’ recommendations than control group parents. This effect was evident at 1-month postintervention and 3-month follow-up, with small to medium effect sizes. Additionally, most parents were satisfied with the intervention and found it useful. The intervention did not, however, have a significant effect on any of the secondary outcome measures; namely adolescent-report of parenting, or symptoms of depression or anxiety in the adolescents. Our findings therefore do not support the effectiveness of the intervention for reducing adolescent symptoms in the short-term.

### Effect of the Intervention on Parenting

The current findings add to a growing body of literature supporting the efficacy of preventive parenting interventions for the improvement of modifiable parenting factors (eg, [33,35,82-84]). This study contributes a number of novel findings to the literature. To our knowledge, the intervention is the first of its kind to target a wide range of parenting factors known to influence the development of depression and anxiety disorders in adolescents. Additionally, the intervention is considerably briefer than existing parenting interventions and was conducted entirely online with no therapist support. It is therefore promising to find an improvement in self-reported parenting, particularly given that many existing interventions are labor-intensive (eg, delivered by trained professionals) and expensive to disseminate. Together, these factors support the potential of a single-session, online intervention as a strategy to translate research regarding parenting risk and protective factors for adolescent depression and anxiety into an accessible, low-cost preventive approach. Importantly, this study also adds to the paucity of research investigating preventive interventions for parents of adolescents.

Although we found a significant effect of the intervention on self-reported parental concordance with the Guidelines, there was no significant effect of the intervention on adolescent-report of parenting. In fact, adolescents in both groups reported a slight reduction in their parent’s concordance with the Guidelines’ recommendations. This discrepancy is in line with the frequently reported discordance between parent- and child-report of parenting (eg, [85-87]). It is also reflected by the modest correlation between the PRADAS and PRADAS-A (*r*=.25), which is consistent with associations between parent and child report in the literature (eg, correlations of .20 to .40 are typical [85,86,88]).

There are several possible explanations for the discrepancy between parent- and adolescent-report found in this study. First, the perspectives captured by parent- and child-report may reflect the different focus or importance that parents and adolescents place on various parenting practices. For example, parents may report on subtle behaviors, attitudes, and beliefs that may not be noticed by the adolescent. Similarly, adolescents’ reports may be influenced by their experiences over a longer period of time, rather than specific recent parenting behaviors. Second, the PRADAS-A covers less content and is less specific than the PRADAS, with fewer items (43 compared with 73 items of the PRADAS). It may therefore be less sensitive to detect subtle or gradual changes in parenting, which may not be easily detected by adolescents, particularly in the short-term. As this is the first study to utilize the PRADAS-A, we do not have prior evidence to support its ability to detect change. It is also possible that adolescents in our sample, who had a mean age of 13.64 years, were too young to accurately report change in the parenting practices covered in the PRADAS-A. Finally, it could be that our results only demonstrate parents’ perceived improvement in parenting, as measured by the self-report PRADAS. Without objective measures of behavior and with the discrepancy between respondents, we cannot be certain that the intervention did result in improved parenting practices. Unfortunately, this is a limitation inherent in many parenting intervention studies, which often use single-informant parent-report measures of family and parenting factors.

### Effect of the Intervention on Adolescent Symptoms

The lack of effect of the intervention on adolescent symptoms contrasts with a growing body of literature suggesting that preventive parenting interventions can reduce internalizing problems in children and adolescents (eg, [31,32,35,37,89]). There are a number of potential reasons for this finding. First, we only collected data up to 3-month follow-up. It is likely that a longer time period is required for changes in parenting to influence adolescent symptoms. Much of the evidence base for the parenting factors targeted in the intervention stems from longitudinal research [9]; hence, the parenting factors are theorized to have a long-term impact on adolescent outcomes. For example, factors such as consistent discipline or supporting age-appropriate autonomy would likely take time to have an effect on adolescent symptoms. In line with this hypothesis, several studies that have found beneficial effects of parenting interventions on child mental health outcomes have had longer follow-up periods (eg, 6-12 months or more; see [37,84] for reviews) or have found that effects increase over time (eg, [29,30,90]). It is possible that long-term effects of preventive parenting interventions occur because of “sleeper effects.” That is, the effects of the intervention on adolescent outcomes may increase over time, potentially because of the bidirectional nature of parenting behaviors and child temperament or behavior on subsequent child internalizing outcomes (eg, [14,37,91,92]). To adequately assess the relationship between improving parenting factors and adolescent depression and anxiety outcomes, longer-term studies, ideally with follow-up into late adolescence, are required.

It should also be noted that although there is strong evidence regarding the role of parenting in the development of child and adolescent depression and anxiety, the amount of variance explained by individual parenting factors is small (eg, [9,38-40]). The development of these disorders is multifaceted and influenced by many factors other than parenting (eg, gender, genetic predisposition, parent psychopathology, early-life events, and socioeconomic factors [10,93-95]), as well as interactions among these factors [93,95]. In line with this, the correlations between the PRADAS and adolescent symptoms at baseline were small (SCAS-P: *r=* −.13, *P*=.02; SCAS-C: *r*=−.09, *P*=.10; SMFQ-P: *r*=−.22, *P*<.001; SMFQ-C: *r*=−.11, *P*=.06). It is therefore possible that the change in parenting found in this study was not large enough to result in reduced adolescent symptoms in the short-term. Additionally, to ensure independence of observations, we only allowed one parent per family to participate. This differs to face-to-face parenting interventions, which typically invite both parents to participate (although participation of mothers is unequivocally higher [96]), and emphasize the importance of parenting consistency. Thus, while changing the parenting of one parent was not effective in reducing adolescent symptoms in this study, it is possible that a consistent change across both parents could have greater influence on adolescent outcomes.

### Implications for Universal Prevention

Evidence to date is conflicting regarding the comparative efficacy of universal, selective, and indicated prevention approaches for depression and anxiety in young people. Some reviews have found selective or indicated programs to be more effective (eg, [28,97-99]), whereas others have found no significant differences between the approaches (eg, [23,37,100]). Although effect sizes may be larger in selective and indicated programs, universal approaches have the benefit of reaching a greater proportion of the population. Thus, even with small effect sizes, the population mean can be shifted, resulting in significant impact at a population level [101]. The small effect on parent-reported behaviors found in this study could therefore lead to population-level changes in parenting if the intervention is delivered universally.

In practice, the optimal plan for dissemination of preventive parenting programs for adolescent depression and anxiety would likely involve a combination of universal, selective, and indicated approaches [93]. In this way, universal programs are able to reach a greater proportion of the population, whereas selective and indicated programs, which are typically more intensive and costly to deliver, are available for those at elevated risk. As discussed, this is the aim of the multilevel PiP program [58]. The single-session intervention evaluated in this paper can serve as a first step, which can be accessed by all parents regardless of their adolescent’s risk or current symptoms. This may have the added benefit of minimizing stigma, if promoted as a resource for all parents of adolescents (eg, at the transition to secondary school). Given the difficulty engaging and retaining parents in online interventions, a model such as this may allow more parents to receive at least some intervention (ie, the single-session component), even if they do not continue to complete the PiP modules. Parents then have the option of “stepping up” to the next level of PiP based on personal preference or need. Results from this study support the value of including the single-session component as a stand-alone intervention within the PiP model. In comparison to a recent RCT comparing the full PiP intervention with an active control condition, the single-session intervention achieved a similar effect at 3-month follow-up (although the overall effect size was larger for the full intervention [65]).

### Strengths, Limitations, and Future Directions

This study has several strengths. We recruited a large community sample via methods similar to how the intervention could be disseminated (ie, primarily via schools and online networks). The demographics of our sample are therefore likely to be representative of the expected end users of the program. Similarly, there were no exclusion criteria, and participants were not precluded from accessing other resources or services. These factors suggest good external validity of the findings. Furthermore, parents were generally satisfied with the intervention and found the feedback to be useful. This may partially account for the low attrition rate, which is notable given the high attrition often reported in online interventions (eg, [63,66,67]).

Study limitations include the overrepresentation of highly educated mothers from intact families, which may limit the generalizability of findings to fathers, parents with lower educational attainment, and different family situations. Although not representative of the general population, our sample characteristics are similar to other studies of online parenting interventions (eg, [56,57,102,103]), suggesting that these types of programs may be most appealing to this demographic. This limitation is particularly relevant given the aim of promoting the single-session intervention (and PiP more broadly) as a universal prevention approach. The impact of the intervention at a population level would be limited if it is only accessed by a certain demographic, particularly given that parent educational attainment is associated with higher positive parenting practices [59,104,105] and lower rates of child mental health problems [106,107]. To adequately assess the potential of the intervention as a universal approach, research with more diverse samples is required. Our team is currently planning a trial of PiP with parents of lower socioeconomic status. It is possible that parents with more risk factors (ie, lower concordance with the Guidelines’ recommendations) may show greater improvement with intervention, as they have more room for improvement. Conversely, the single-session approach may not be a sufficient level of intervention for such parents, although it may be a useful way to initially engage or identify parents who could benefit from a more intensive parenting support program.

We assessed parenting with newly developed measures, designed specifically to assess concordance with the Guidelines. This was necessary to assess the wide range of parenting factors covered in the intervention with minimal burden to participants. However, it was in place of existing validated measures; therefore, future research is required to assess whether there are effects of the intervention beyond what is captured by the PRADAS. Although not feasible in this study, the addition of objective measures of parenting, such as behavioral observation of parent-adolescent interactions, would also be of value in future research. Additionally, we only collected data up to 3-month follow-up, and the ethical need to limit the duration of waitlist precludes the possibility of between-group comparison of long-term follow-up. We also used symptom measures, rather than measures of diagnostic status. Future research would benefit from including diagnostic measures, as well as broader measures of quality of life and general functioning. Examining the effectiveness of the program when both parents participate would also be of value.

### Conclusions

This RCT provides preliminary support for the effectiveness of a single-session, Web-based, tailored psychoeducation parenting intervention for improving evidence-based parenting risk and protective factors for adolescent depression and anxiety disorders. At this stage, there is no evidence that the program reduces symptoms of depression and anxiety in adolescents in the short-term. However, given the empirical and theoretical basis for the parenting factors targeted in the intervention, it is possible that altering these parenting behaviors in early adolescence could result in reduced risk in the long-term. Given the brevity and ease of dissemination of the program, this is a promising avenue for the translation of research into a sustainable, low-cost intervention that can be disseminated widely as a public health prevention strategy.

## Appendix

Multimedia Appendix 1 Screenshots of the intervention.

Multimedia Appendix 2 Parental responses to process evaluation questions.

Multimedia Appendix 3 Parent explanatory statement.

Multimedia Appendix 4 Child explanatory statement.

Multimedia Appendix 5 Online registration and consent form.

Multimedia Appendix 6 Observed scores for primary and secondary outcome measures at each measurement occasion.

Multimedia Appendix 7 CONSORT-EHEALTH Checklist (V 1.6.1).

## Abbreviations

RCT: randomized controlled trial
PRADAS: Parenting to Reduce Adolescent Depression and Anxiety Scale (-A: Adolescent report)
SCAS: Spence Children’s Anxiety Scale (-C: Child report; -P: Parent report)
SMFQ: Short Mood and Feelings Questionnaire (-C: Child report; -P: Parent report)
MMRM: Mixed effect Model Repeated Measures
PiP: Partners in Parenting
