# Supplementary material for: A Single-Session, Web-Based Parenting Intervention to Prevent Adolescent Depression and Anxiety Disorders: Randomized Controlled Trial
Source: J Med Internet Res. 2018 Apr 26;20(4):e148. doi: 10.2196/jmir.9499 (PMC5945988; doi:10.2196/jmir.9499)
Supplement: Multimedia Appendix 1 [file jmir_v20i4e148_app1.pdf]

Multimedia Appendix 1. Screenshots of the intervention

(a) Parenting to Reduce Adolescent Depression and Anxiety Scale (PRADAS)
(b) Personalised parenting feedback

(a)

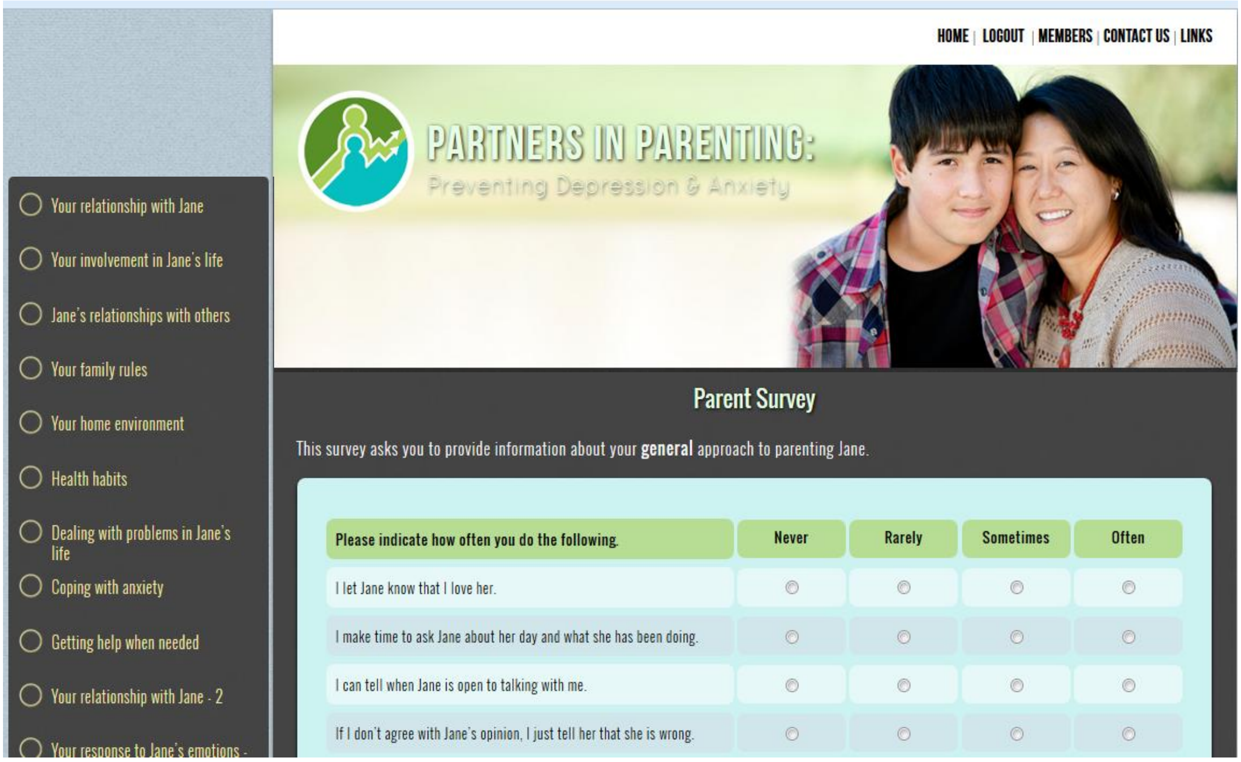

(b)

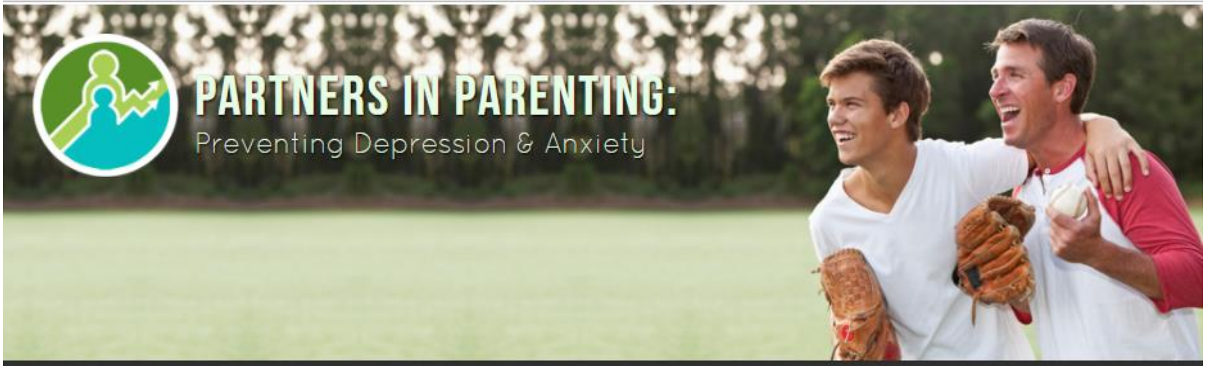

Thank you for completing the survey!

We can now provide you with feedback about your role in reducing Sam's risk of developing depression and clinical anxiety. We also provide you with some practical strategies that you could use to further support Sam. A copy of this feedback has also been sent to your email address.

What next?

This feedback report contains a number of strategies that may be useful for you and Sam. This may seem like a lot of information to take in at once. If you're not sure where to start, you may like to have a quick read over it first. You don't need to try all of the recommended strategies at once. Remember, change can take time and patience. If you feel that there are things you could have done differently as a parent, try not to be too hard on yourself. You've already taken a positive step by completing the survey, well done!

Click on a title to expand your feedback.

Your Relationship with Sam

Your Involvement in Sam's Life

It is important to find a balance between being involved in Sam's life and giving him age-appropriate independence. Teenagers benefit most when their parents continue to show interest in their lives without being intrusive, and respect their need for growing independence.

You can be more involved in Sam's life by:

- Showing an interest in what Sam does at school. For example, you could ask him what he did during the day, what his favourite subjects are, or which teachers he likes.
- If Sam is going out without you, have a casual chat with him about what he will be doing, where he will be, and who he will be with.

You can further support Sam's growing independence by:

- Encouraging Sam to try out extra-curricular activities (e.g. sports, music, or anything else he is interested in).
- Allowing Sam to become more independent of you over time. Evaluate whether you are taking over things too much. For example, you can ask yourself, "Did I really need to step in?" and "What would have been the worst thing to happen if I didn't step in?"
- Gradually increasing Sam's responsibilities and independence over time to allow him to mature.

Sam's Relationships with Others

Your Family Rules

Your Home Environment

Health Habits

Dealing with Problems in Sam's Life

Coping with Anxiety

Getting Help When Needed
