# Supplementary material for: A Single-Session, Web-Based Parenting Intervention to Prevent Adolescent Depression and Anxiety Disorders: Randomized Controlled Trial
Source: J Med Internet Res. 2018 Apr 26;20(4):e148. doi: 10.2196/jmir.9499 (PMC5945988; doi:10.2196/jmir.9499)
Supplement: Multimedia Appendix 2 [file jmir_v20i4e148_app2.pdf]

Multimedia Appendix 2. Parental responses to process evaluation questions

Table 1. Frequencies of parental responses to process evaluation questions administered after receiving the intervention

Question	N	%
**How much of your feedback have you read at this stage? (n=265)**		
All of it	206	77.7
About half of it	51	19.2
I will only read it later	8	3.0
I'm not interested in it	0	0.0
**How satisfied are you with the feedback you received about your parenting? (n=264)**		
Very satisfied	129	48.9
Somewhat satisfied	118	44.7
Somewhat dissatisfied	14	5.3
Very dissatisfied	3	1.1
**How useful did you find the feedback you received about your parenting? (n=264)**		
Extremely useful	45	17.0
Very useful	111	42.0
Somewhat useful	95	36.0
Not useful	13	4.9
**How likely are you to change your parenting based on this feedback? (n=264)**		
Very likely	92	34.8
Somewhat likely	146	55.3
Somewhat unlikely	19	7.2
Very unlikely	7	2.7
**How confident are you in your ability to change your parenting based on this feedback? (n=264)**		
Very confident	98	37.1
Moderately confident	124	47.0
A little confident	35	13.3
Not at all confident	7	2.7

Note. Percentages reflect the proportion of participants who answered these items. Reduced Ns are due to approximately 24% of participants declining to answer these items.
