# Supplementary material for: A Single-Session, Web-Based Parenting Intervention to Prevent Adolescent Depression and Anxiety Disorders: Randomized Controlled Trial
Source: J Med Internet Res. 2018 Apr 26;20(4):e148. doi: 10.2196/jmir.9499 (PMC5945988; doi:10.2196/jmir.9499)
Supplement: Multimedia Appendix 3 [file jmir_v20i4e148_app3.pdf]

MONASH University

PARENT EXPLANATORY STATEMENT

Partners in Parenting: Evaluating a Brief Online Parenting Program

Chief Investigator: Dr Marie Yap, School of Psychological Sciences, Faculty of Medicine, Nursing and Health Sciences, Monash University. Phone: (03) 9905 0723, email: marie.yap@monash.edu
Student Researchers: Mairead Cardamone-Breen, Doctor of Clinical Psychology candidate; Peter Martin, Honours student, Brooke Swierzbiolek, Honours student; School of Psychological Sciences, Faculty of Medicine, Nursing and Health Sciences Monash University. Phone: (03) 9905 1250, email: med-parentingstrategies@monash.edu
Senior Research Officer: Shireen Mahtani, School of Psychological Sciences, Monash University. Phone: (03) 9905 1250, email: med-parentingstrategies@monash.edu

Invitation to participate in research
You are invited to take part in research being conducted by Monash University. Please read this Explanatory Statement in full before deciding whether or not to participate. If you would like further information regarding any aspect of this project, please contact the researchers via the phone numbers or email addresses listed above.

What does the research involve?
This study aims to evaluate the effects of a brief, individually-tailored web-based parenting program to reduce the risk of depression and anxiety disorders in teenagers (aged 12 to 15 years). The program is designed to provide parents with personalised feedback about their current parenting, including what they are doing well and areas that they can improve in order to reduce their teenager's risk of depression and anxiety. We are interested to see whether this program can help improve parenting behaviours, and reduce risk of depression and anxiety in teenagers.

What will I be asked to do?
If you choose to participate, you will be asked to do the following:

1. Go online to http://parentingstrategies.net/depression.brief.intervention/index.php
   Fill in the online registration and consent form, and indicate that you have discussed the project with your child and that they agree to be contacted by a member of the research team. You will also be asked to provide some basic demographic information so that we can ensure you are eligible for this study. We will also ask you to provide a telephone number that we can contact your child on at a preferred time and day of the week. If you consent for both you and your child to participate, click on 'Register us for this study'.
2. Your child will be contacted by a member of the research team from the Monash University School of Psychological Sciences. The purpose of this phone call is to discuss the study with your child and ensure that they understand what will be involved if they agree to take part. If your child agrees to participate, the researcher will give them their own login details, and provide any guidance to them as needed over the phone, to complete their online assessment. This online assessment includes questions about your child's experience of your current parenting practices, and different feelings and behaviours that may be associated with depression and anxiety (30-45 minutes altogether). Your child will be asked to complete the same online assessment again 3 months later.
3. You will then receive an email requesting that you complete an online assessment asking about your current parenting practices regarding your child, as well as questions about feelings and behaviours in your child that may be associated with depression and anxiety (50-65 minutes altogether).
4. You will then be randomly allocated into one of two groups, where you will receive either:
   a. A tailored feedback report about your parenting based on your survey responses as well as the parenting guidelines *How to prevent depression and clinical anxiety in your teenager: Strategies for parents,* **immediately** after completing your first survey; or
   b. A tailored feedback report about your parenting based on your survey responses as well as the parenting guidelines *How to prevent depression and clinical anxiety in your teenager: Strategies for parents,* immediately after completing your follow-up survey **3 months later.**

Allocation to each group will be automated by a computer program. You will find out which group you have been allocated to after you complete your first online assessment.

5. You will be contacted again 1 month later to complete a similar online survey (35-50 minutes).
6. You and your child (if they agree to take part) will be contacted again approximately 3 months later to complete similar online surveys (30-45 minutes each).
7. If you are allocated to the first group above, you and your child (if they agree to take part) will be contacted again 6 and 12 months after your first online assessment, and be invited to complete similar online surveys (30-45 minutes each).

All parts of this study can be completed at any time or place of convenience to you, as long as there is internet access.

Who is being asked to participate?

We are inviting families with at least one child aged between 12 and 15 years (inclusive), who live in Australia and are proficient in English, to participate in this research. One parent and one child (aged 12-15) from each family are invited to participate. Participants also need regular access to the internet.

Our parenting program is designed for *preventing* teenage depression and anxiety disorders. As such, if you think that your teenager may need professional treatment for their difficulties with depression or clinical anxiety, we recommend that you see a trained mental health professional instead of participating in this research. If you and your child indicate in your survey responses that your child may be having difficulties with their mood or anxiety, we will get in touch with both of you to discuss how you may access further support for your child as needed.

Source of funding

This research project has been funded by Monash University.

What if I change my mind?

Participation in this research is completely voluntary. If you decide to participate, you will be asked to provide consent for yourself and your child, by completing an online registration and consent form. We will also check that your child is happy to participate, over the phone. If you or your child change your mind at any stage, you are free to withdraw from the research. You may also request that your data (i.e. survey responses) be withdrawn once submitted, prior to the final report being written. After this time, you will not be able to withdraw your data.

What are the benefits?

This project is designed to help equip parents with parenting strategies that can help reduce the risk of depression and anxiety in their teenagers. You will be provided with tips for making changes to your parenting that may protect your child against these problems. In the longer term, it is hoped that parents who use the program will benefit by helping to reduce their teenager's risk of depression and anxiety problems.

Are there any risks?

The risks of participating in this research are low, however we have identified the following potential risks:

1. Although unlikely, it is possible that you or your child may become upset while completing the surveys.
2. Although unlikely, it is possible that you may become distressed by the feedback you receive on your current parenting practices.

If you do become distressed at any stage, please seek support if you feel you need to talk to someone about how you are feeling. You may have supports already available (e.g. family, friends, health professionals). Otherwise, you can call one of the help-line numbers listed below, or see details of where you can seek help on the website http://www.parentingstrategies.net/depression/links.php

- ***Lifeline: 13 11 14 (24 hours a day, 7 days a week)***
- ***Parentline: 13 22 89 (8am – midnight, 7 days a week)***

3. Although unlikely, there is also a risk to your privacy through breaches of confidentiality, particularly if there is a risk of harm to yourself or others that cannot be prevented without breaching confidentiality. If we believe that yourself, your child, or someone else is at risk of harm (e.g. child abuse, self-harm) we are bound by professional codes of ethics to take reasonable action to prevent this harm occurring, even if these means breaching confidentiality. If this is necessary, we will discuss the situation with you (and your child, if appropriate).

Reimbursement
To thank you for your time and commitment to our research, you will receive a $10 e-gift voucher after completing your 3-month follow-up survey. If you are allocated to the first group above, you will also receive a $10 e-gift voucher after completing each of your 6 and 12 month follow-up surveys. Your child will receive a $10 e-gift voucher after completing their initial survey and again after their 3-month follow-up survey. If you are allocated to the first group above, your child will also receive another $10 e-gift voucher after completing each of their 6 and 12-month follow-up surveys. Reimbursement will be in the form of Coles e-gift vouchers for yourself, and either Coles or iTunes e-gift vouchers for your child. Vouchers will be emailed separately to you and your child.

What about my privacy?
All data collected will be stored in a secure online database, and will be kept separate to any identifying information, to protect your privacy. Information obtained in the research will only be accessible by the researchers named on the project. All electronic files will be password protected. All data will be securely destroyed after a minimum of 5 years from when the final report of the study is published. Any written reports will only include group data, and will not be identifiable in any way.

How can I see the results?
A summary of results will be available in 2017. If you would like a copy, please contact Mairead Cardamone-Breen (mairead.cardamone-breen@monash.edu). Results will be included in the Doctoral and Honours theses of the student researchers, and may be presented at conferences. We also aim to have the research published in scientific journals.

Use of data for other purposes
If you consent, the information you and your child provide during this study may be used for other research purposes, in a non-identifiable form. Such future studies will be subject to approval from the relevant Ethics Committee. You can choose not to have you or child's data included in future research if you wish.

What if I have any complaints or concerns?
This project has been approved by the Monash University Human Research Ethics Committee. Should you have any concerns or complaints about the conduct of the project, you are welcome to contact:

Executive Officer
Monash University Human Research Ethics Committee (MUHREC)
Room 111, Building 3e
Research Office
Monash University, Clayton, VIC, 3800
Tel: +61 3 9905 2052 Email: muhrec@monash.edu Fax: +61 3 9905 3831

Thank you,

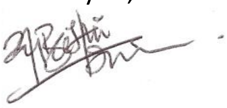

Dr Marie Yap
NHMRC Career Development Fellow
Senior Research Fellow and Psychologist
School of Psychological Sciences
Monash University
