# Supplementary material for: A Single-Session, Web-Based Parenting Intervention to Prevent Adolescent Depression and Anxiety Disorders: Randomized Controlled Trial
Source: J Med Internet Res. 2018 Apr 26;20(4):e148. doi: 10.2196/jmir.9499 (PMC5945988; doi:10.2196/jmir.9499)
Supplement: Multimedia Appendix 4 [file jmir_v20i4e148_app4.pdf]

MONASH University

CHILD EXPLANATORY STATEMENT

Partners in Parenting: Evaluating a Brief Online Parenting Program

Chief Investigator: Dr Marie Yap, School of Psychological Sciences, Faculty of Medicine, Nursing and Health Sciences, Monash University. Phone: (03) 9905 0723, email: marie.yap@monash.edu
Student Researchers: Mairead Cardamone-Breen, Doctor of Clinical Psychology candidate; Peter Martin, Honours student, Brooke Swierzbiolek, Honours student; School of Psychological Sciences, Faculty of Medicine, Nursing and Health Sciences Monash University. Phone: (03) 9905 1250, email: med-parentingstrategies@monash.edu
Senior Research Officer: Shireen Mahtani, School of Psychological Sciences, Monash University. Phone: (03) 9905 1250, email: med-parentingstrategies@monash.edu

Invitation to participate in research
You are invited to take part in research being conducted by Monash University. Please read this Explanatory Statement before deciding whether or not to participate. If you would like more information, please contact the researchers via the phone numbers or email addresses above.

What is the research about?
We are interested in how parents can help protect their teenagers from developing problems with depression and anxiety. We want to find out whether a new online program can help parents to do things that might protect their children from these problems.

What will I be asked to do?
If you choose to take part, one of the researchers will call you (at a time that suits you) to check that you understand what we are asking you to do, and so that you can ask any questions. We will then ask you to complete an online survey (a researcher will be on the phone to help you do this, if needed). The survey will ask you questions about your parents (e.g. your relationship with them, the things you do together) and also about your feelings and behaviours that may be linked to depression and anxiety (e.g. whether you feel sad or worried about things). The survey will take around 30-45 minutes.
We will ask you to do a similar survey 3 months later. We may also contact you again after 6 and 12 months, and ask you to do another 2 surveys. All of the surveys can be completed whenever you like, as long as you have internet access.

Who is being asked to take part?
We are asking young people, aged 12 to 15 to be a part of this study, together with one of their parents. You need to live in Australia, speak English, and have access to the internet.

Source of funding
This research project has been funded by Monash University.

What if I change my mind?
If, for any reason, you don't want to be a part of this study anymore, you can pull out at any time. No-one, even your parents, can make you continue if you don't want to.

What's in it for me?
There may not be any direct benefit for you taking part in this study, but you will be helping us to find out whether this new online parenting program can reduce the risk of depression and anxiety in young people.
To thank you for your time, you will receive a $10 e-gift voucher after you do your first survey, and another $10 e-gift voucher after you complete the survey again 3 months later. If we contact you again after 6 and 12 months, you

will get another $10 e-gift voucher after completing each of these surveys. You can choose between an iTunes or Coles e-gift voucher (sent to your preferred email address).

Are there any risks to my parent or me?
We don't think there are any major risks of taking part in this study. However, it is possible that you may be upset by some of the questions in the survey. If you do feel upset at any stage, you can call the **Kids' Helpline on 1800 55 1800** to speak to a counsellor. You can call at any time, any day, and there will be someone available to talk to you, for free.

Will other people find out what I say in the surveys?
Everything you tell us as part of this research will be kept private. Even your parents won't be able to find out what you say in the surveys. After you have done the surveys, we will separate your name and other information that can identify you by giving you an ID number. All your information will be password protected.
We will only tell your parents or another adult what you have told us if we think that you or someone else is likely to be hurt. Also, if you become upset while we are on the phone to you, we will ask to speak to your parent to discuss what they can do to support you.
If we think that you may be having some problems with depression and anxiety (based on what you tell us in the surveys), we may suggest to your parents that they take you to a professional who can help you with these problems.

What if I have any complaints or concerns?
This project has been approved by the Monash University Human Research Ethics Committee. Should you have any concerns or complaints about the conduct of the project, you are welcome to contact:

Executive Officer
Monash University Human Research Ethics Committee (MUHREC)
Room 111, Building 3e
Research Office
Monash University, Clayton, VIC, 3800
Tel: +61 3 9905 2052 Email: muhrec@monash.edu Fax: +61 3 9905 3831

Thank you,

Dr Marie Yap
NHMRC Career Development Fellow
Senior Research Fellow and Psychologist
School of Psychological Sciences
Monash University
