# Supplementary material for: A Single-Session, Web-Based Parenting Intervention to Prevent Adolescent Depression and Anxiety Disorders: Randomized Controlled Trial
Source: J Med Internet Res. 2018 Apr 26;20(4):e148. doi: 10.2196/jmir.9499 (PMC5945988; doi:10.2196/jmir.9499)
Supplement: Multimedia Appendix 5 [file jmir_v20i4e148_app5.pdf]

Multimedia Appendix 5. Online Registration and Consent Form

To find out more about the study, please read the following Explanatory Statements (see attached).

By completing this Registration and Consent Form, you are telling us that:

☐ You understand what you have read in the Parent Explanatory Statement

☐ You understand what you have read in the Child Explanatory Statement and

☐ You provide consent for yourself and your child to take part in this research study

Please note, as our program is designed to prevent depression and anxiety disorders in teenagers, it is not suited for parents who think their teenager may be in need of professional treatment for mental health concerns. If your teenager is currently having difficulties with depression or anxiety, we recommend that you see a trained mental health professional rather than participating in this research.

- **Parent Information**
- First Name:
- Surname:
- Age:
- Gender:
- Postcode:
- Preferred tel:

- Alternate tel:
- Preferred contact number for your child:
- What is your highest level of education?
- Are you currently employed?
- Are you currently studying?
- Do you speak a language other than English at home?
- Are you Aboriginal or Torres Strait Islander?
- **Registration Information**
- Email:
- Password:
- Confirm password:
- **Your Family's Characteristics**
- What is your marital status?
- How would you describe your family situation?

- How many children do you have living with you?

- We would like to find out why people are interested to participate in a study providing parenting strategies for preventing adolescent depression and anxiety. Can you please tell us briefly any reasons you have for wanting to participate?

-

- **Child Information**
- To be eligible to participate, your child must be aged between 12 and 15.
- First Name:
- Surname:
- Date of birth: Day Month Year
- Gender:
- What is your relationship to this child?
- Who does this child live with?

- Is there a preferred day/time in the next 2 weeks, to call your child about the study? Preferably Monday to Friday, before 7:30pm. For example, Wednesdays 4 -7pm.

- As a parent, I understand that my participation will involve being randomly placed into one of the following two groups where I will receive:
    1. A tailored feedback report about my parenting based on my survey responses as well as the parenting guidelines *How to prevent depression and clinical anxiety in your teenager: Strategies for parents*, immediately after completing my first survey; or
    2. A tailored feedback report about my parenting based on my survey responses as well as the parenting guidelines *How to prevent depression and clinical anxiety in your teenager: Strategies for parents*, immediately after completing my follow-up survey 3 months later.
- I understand that only one of my children can take part in this study.
- I have spoken with my child about participating in this study and explained the level of commitment that would be expected from them, and my child is willing to take part. I understand that if my child does not wish to participate in this study, I will not be able to take part either.
- I understand that my child's phone call and online survey will take approximately 30 minutes and my online survey will take approximately 60 minutes. I will be contacted by email again after one month to complete a similar online survey. Thereafter, both my child and I will be invited to complete surveys of similar length after 3 months.
- If I am randomly placed in group 1 above, I understand that both my child and I will be invited to complete surveys of similar length 6 and 12 months after our first surveys.
- I also understand that we are free to withdraw our consent and discontinue our participation at any time without explanation. We would also be able to withdraw any unprocessed identifiable data previously supplied.
- Our decision whether or not to participate will not prejudice any future relationship we may have with Monash University or The University of Melbourne.
- I understand that all information provided by my child and I will be kept strictly confidential within the limits of the law (i.e., unless the researchers find evidence of any offence that must be reported to authorities by law), and will be accessible only by researchers involved in this study.
- I agree that I will not be informed about any of my child's responses to the surveys, unless my child reveals that they or someone else is likely to be hurt.
- I agree that research data gathered from the results of the study may be published, provided that we cannot be identified.

Any concerns about the scientific aspects of the study can be directed to Dr Marie Yap via email, marie.yap@monash.edu

Any complaints about the ethical aspects of the research may be directed to the Executive Officer, Monash University Human Research Ethics Committee (MUHREC), Monash University, Clayton, Vic 3800, email: muhrec@monash.edu, phone 03 9905 2058, fax 03 9347 6739.

Button that says:

**Both my child and I agree to participate in this study**

Participant must click on button to proceed to the next page.
