# Supplementary material for: A Single-Session, Web-Based Parenting Intervention to Prevent Adolescent Depression and Anxiety Disorders: Randomized Controlled Trial
Source: J Med Internet Res. 2018 Apr 26;20(4):e148. doi: 10.2196/jmir.9499 (PMC5945988; doi:10.2196/jmir.9499)
Supplement: Multimedia Appendix 6 [file jmir_v20i4e148_app6.pdf]

Multimedia Appendix 6. Observed scores for primary and secondary outcome measures at each measurement occasion

Table 1. Observed means, standard deviations, minimum and maximum for PRADAS at baseline, 1-month and 3-month follow-up

**Group**	**Occasion**		
	**Baseline**	**1-month follow-up**	**3-month follow-up**
**Intervention**			
M (SD)	46.94 (7.86)	51.18 (7.55)	51.83 (8.14)
Minimum, maximum	22, 64	31, 67	23, 68
n	164	153	151
**Control**			
M (SD)	47.81 (6.68)	49.35 (6.03)	49.81 (6.19)
Minimum, maximum	28, 62	34, 65	26, 63
n	185	176	177

Table 2. Observed means, standard deviations, minimum and maximum for all secondary outcome measures at baseline and 3-month follow-up.

Measure	Intervention		Control	
	Baseline	3-month	Baseline	3-month
**PRADAS-A**				
M (SD)	24.55 (5.52)	23.95 (6.14)	24.46 (5.46)	23.93 (5.96)
Minimum, maximum	9, 34	8, 39	9, 37	7, 37
n	154	146	173	159
**SCAS-P**				
M (SD)	16.65 (10.82)	13.30 (9.90)	16.86 (12.39)	14.04 (11.57)
Minimum, maximum	1, 64	0, 46	1, 97	0, 79
n	164	151	185	177
**SCAS-C**				
M (SD)	30.86 (19.08)	29.85 (18.85)	28.82 (16.73)	26.58 (18.26)
Minimum, maximum	3, 103	0, 99	4, 88	0, 99
n	153	145	173	159
**SMFQ-P**				
M (SD)	4.43 (4.73)	3.29 (4.22)	4.15 (4.45)	3.47 (3.97)
Minimum, maximum	0, 22	0, 24	0, 25	0, 16
n	164	151	185	177
**SMFQ-C**				
M (SD)	6.43 (5.63)	6.59 (6.10)	5.97 (5.70)	5.84 (6.09)
Minimum, maximum	0, 25	0, 25	0, 25	0, 26
n	152	146	173	159

Note. PRADAS-A = Parenting to Reduce Adolescent Depression and Anxiety Scale – Adolescent-report; SCAS-P = Spence Children's Anxiety Scale, Parent-report; SCAS-C = Spence Children's Anxiety Scale, Child-report; SMFQ-P = Short Mood and Feelings Questionnaire, Parent-report; SMFQ-C = Short Mood and Feelings Questionnaire, Child-report.
